# Sulcus-Based MR Analysis of Focal Cortical Dysplasia Located in the Central Region

**DOI:** 10.1371/journal.pone.0122252

**Published:** 2015-03-30

**Authors:** Pauline Roca, Charles Mellerio, Francine Chassoux, Denis Rivière, Arnaud Cachia, Sylvain Charron, Stéphanie Lion, Jean-François Mangin, Bertrand Devaux, Jean-François Meder, Catherine Oppenheim

**Affiliations:** 1 Department of Neuroimaging, Sainte-Anne Hospital Center, Université Paris Descartes Sorbonne Paris Cité, Center for Psychiatry & Neurosciences, UMR 894 INSERM, Paris, France; 2 Department of Neurosurgery, Sainte-Anne Hospital Center, Université Paris Descartes Sorbonne Paris Cité, Paris, France; 3 UNATI, Neurospin, CEA, Saclay, France; 4 Center for Psychiatry & Neurosciences, Sainte-Anne Hospital Center, UMR 894 INSERM/Université Paris Descartes & Laboratory for the Psychology of Child Development and Education, UMR 8240 CNRS/Université Paris Descartes Sorbonne Paris Cité, Paris, France; University Zurich, SWITZERLAND

## Abstract

**Objective:**

Focal cortical dysplasias (FCDs) are mainly located in the frontal region, with a particular tropism for the central sulcus. Up to 30% of lesions are undetected (magnetic resonance [MR]-negative FCD patients) or belatedly diagnosed by visual analysis of MR images. We propose an automated sulcus-based method to analyze abnormal sulcal patterns associated with central FCD, taking into account the normal interindividual sulcal variability.

**Methods:**

We retrospectively studied 29 right-handed patients with FCD in the central region (including 12 MR negative histologically-confirmed cases) and 29 right-handed controls. The analysis of sulcal abnormalities from T1-weighted MR imaging (MRI) was performed using a graph-based representation of the cortical folds and an automated sulci recognition system, providing a new quantitative criterion to describe sulcal patterns, termed sulcus energy.

**Results:**

Group analysis showed that the central sulcus in the hemisphere ipsilateral to the FCD exhibited an abnormal sulcal pattern compared with controls (p = 0.032). FCDs were associated with abnormal patterns of the central sulci compared with controls (p = 0.006), a result that remained significant when MR-negative and MR-positive patients were considered separately, while the effects of sex, age and MR-field were not significant. At the individual level, sulcus energy alone failed to detect the FCD lesion. We found, however, a significant association between maximum z-scores and the site of FCD (p = 0.0046) which remained significant in MR-negative (p = 0.024) but not in MR-positive patients (p = 0.058). The maximum z-score pointed to an FCD sulcus in four MR-negative and five MR-positive patients.

**Conclusions:**

We identified abnormal sulcal patterns in patients with FCD of the central region compared with healthy controls. The abnormal sulcal patterns ipsilateral to the FCD and the link between sulcus energy and the FCD location strengthen the interest of sulcal abnormalities in FCD patients.

## Introduction

Focal cortical dysplasias (FCDs) are highly epileptogenic lesions due to abnormal neuroglial proliferation and cortical organization [1,2]. They represent one of the main causes of extra-temporal medically refractory yet surgically curable epilepsy. FCDs are predominantly located in the frontal lobe, with a particular tropism for the central region [3]. In the presurgical work-up, accurate detection of the lesion by magnetic resonance imaging (MRI) is crucial, with a better postoperative outcome when MRI findings are positive [4]. MRI features typical of FCD include abnormalities of the cortex (thickening, T2 signal increase, gyral abnormalities) and of subcortical white matter (blurring of the gray–white matter junction, T2 signal increase, “transmantle” sign) [3,5–9]. However, despite advanced MRI protocols, up to 30% of FCDs are not detected by conventional visual analysis [3,10,11]. These patients (hereafter referred to as “MR-negative” [MR-]) may be excluded from surgery, especially if the lesion is suspected to involve highly functional areas such as the primary motor cortex.

Current computer-aided diagnosis tools are mostly based on a subset of the MR criteria typical of FCD (cortical thickening and blurred gray–white matter junction) analyzed with advanced MRI post-processing such as voxel-based morphometry [12–15]. These methods have mainly been tested in patients with FCD detectable by conventional visual analysis (hereafter referred to as MR-positive [MR+]). Additional computational models of FCD, combining analysis of cortical thickness, blurring and tissue intensity derived from T1-weighted imaging, also increase the sensitivity of visual identification of FCD while maintaining a high specificity [16–18].

Unfortunately, when the MR signal of FCD differs only slightly from that of normal tissue, these morphometric methods based on signal intensities may fail. There is therefore a need to look at alternative criteria, such as abnormalities of sulcal morphology [13]. FCDs are developmental lesions, appearing early during cortical maturation and have consequences for the thickness, morphology and gyral organization of the cortex. This results in sulcal abnormalities such as broadening, increased depth, altered orientation [5–7,19] or subclinical abnormal gyration pattern [20]. Recently, a variant sulcal pattern of the central sulcus associated to FCD, named “power button sign” was reported in 62% of a cohort of 37 patients with histologically confirmed FCD of the central region [21]. However, only a few studies performed a quantitative analysis of such sulcal anomalies with a comparison with a group of healthy controls. Besson et al. [22] showed in 43 patients that FCD lesions were preferentially located at the bottom of an abnormally deep sulcus. In addition, Thesen et al. [23] performed a surface-based morphometry analysis of sulcal depth and additional criteria such as local gyrification and curvature in 11 patients (5 with MR+ FCD and other epileptogenic malformations), showing that surface-based morphometry successfully discriminated patients from controls but failed to adequately describe the extent of the lesion in most cases. Recently, automated multivariate supervised classifiers based on surface-based features (sulcal depth, curvature, cortical thickness) and intensity [24] provided a gain in sensitivity over standard radiologic assessment in MR- FCD patients.

In this study, our main aim was to perform a quantitative group-wise analysis of abnormal sulcal patterns associated with FCD in MR- and MR+ patients based on: (1) an automated quantitative analysis of sulcal abnormalities using a novel descriptor of cortical sulci called sulcus energy [20]; (2) three groups of subjects: MR+ patients, MR- patients, and healthy controls; (3) FCD located in the central region, one of the most stable regions in terms of sulcal patterns and a frequent location for FCD. Our second goal was to assess the relevance of sulcus energy z-score maps to localize the epileptogenic lesion at the individual level.

## Methods

### Study groups

We retrospectively studied all consecutive patients referred to our center for intractable epilepsy who fulfilled the following criteria: (1) patients operated from 2000 to 2013; (2) with intractable epilepsy of the central region; (3) and a final diagnosis of FCD, based on typical MRI features, histology and/or on a comprehensive presurgical work-up (including 18-fluorodeoxyglucose positron-emission tomography [PET] and Stereo-EEG). A total of 38 patients fulfilled these criteria. For the purposes of this study we restricted our analysis to the right-handed patients (n = 29). All patients systematically underwent an MR examination as part of their presurgical assessment. FCD lesions were confirmed by histological analysis in 25 of the 29 cases. The remaining 4 patients had typical MRI features making the diagnosis of FCD unquestionable. Twenty-nine right-handed controls without medical history composed the control group. Demographic details of each group are presented in Table 1. These characteristics were similar between groups except for age (p = 0.05 in univariate t-test). This study was approved by the Ethics Review Committee of Ile de France III and has been performed in accordance with the ethical standards laid down in the 1964 declaration of Helsinki and its later amendments. Written inform consent was waived for the patients and was obtained for control subjects in conformity with the laws and regulation of the country in which research experiment was performed (France).

**Table 1:** Demographic data of patients and controls.

	Controls	Patients
Number	29	29
Males (%)	13 (45)	15 (52)
Age: median (IQR)	26 (25–31)	20 (13–29)
Imaged at 3 T (%)	10 (34)	7 (24)

IQR: interquartile range.

### MRI acquisition

Patients and controls were scanned using a 1.5 T (n = 41) or a 3 T (n = 17) MRI scanner (GE Healthcare Medical System, Milwaukee, WI, USA) with a three-dimensional T1-weighted inversion recovery fast spoiled gradient recalled MR pulse sequence. For the patient group, additional T2-weighted and fluid attenuated inversion recovery MR images were acquired as part of their clinical imaging work-up. 3D T1-weighted inversion recovery MR acquisition parameters were as follows: FOV, 220–250 mm; matrix, 256 × 256; slice thickness, 1–1.4 mm; number of slices, 114–146.

### Image post-processing

**Fig 1:**
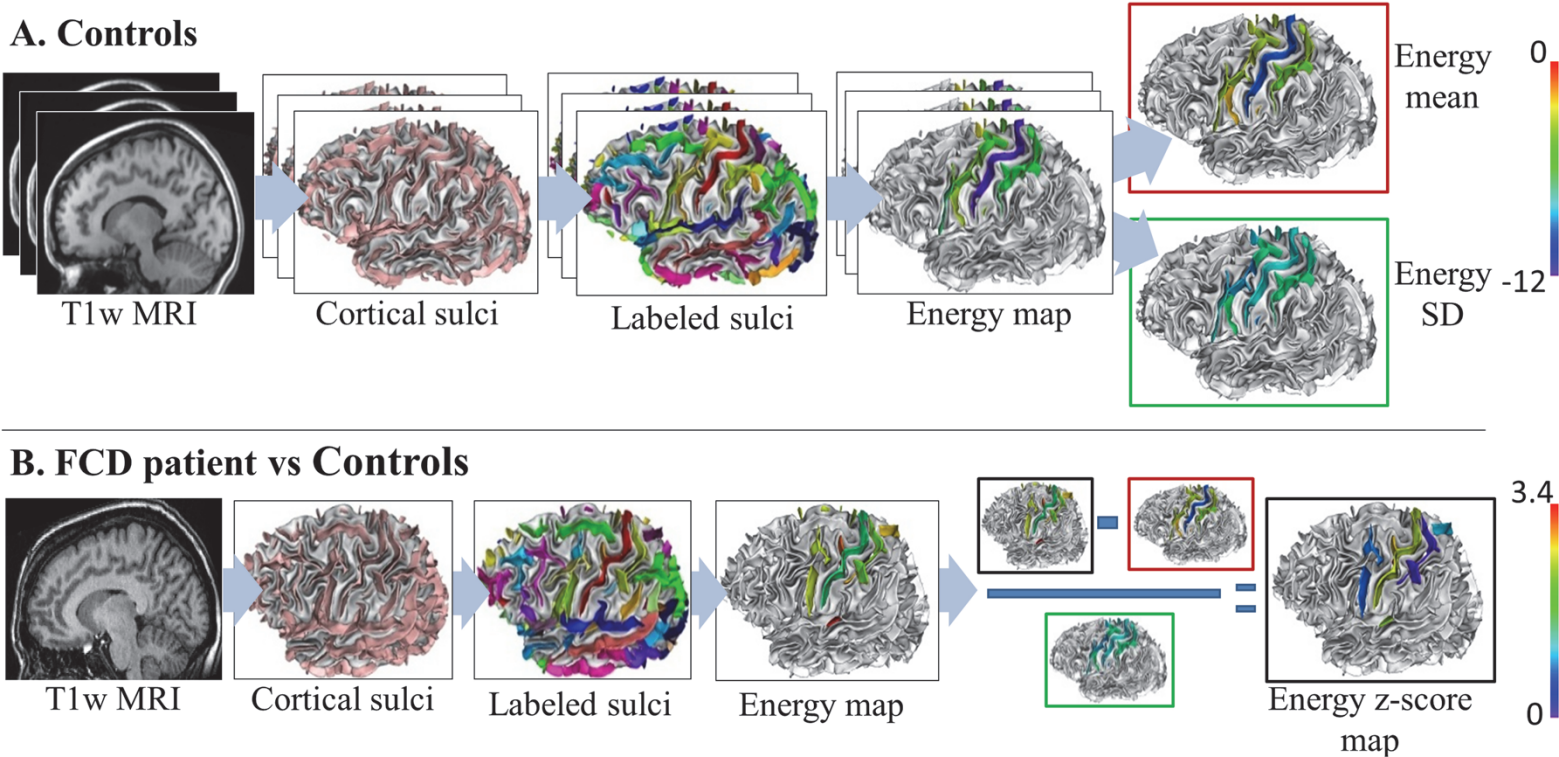
Flowchart of the sulcus-based analysis. A) Processing steps for the controls. First, based on T1-weighted MRI (T1w MRI), cortical sulci were extracted. They were then automatically labeled (one color per label). The sulcus energy maps derived from this recognition process were then generated (blue to red colors, reflecting a sulcus pattern with a good match to the learning database and a bad match, respectively). These maps were averaged to obtain mean and standard deviation (SD) maps. B) Processing steps for a single FCD patient. Cortical sulci extraction, labeling, and generation of sulcus energy map were done as described in A. Finally, a sulcus energy z-score map was computed by dividing the difference between the sulcus energy of the patient and the mean sulcus energy of controls by the standard deviation of controls (blue to red colors, reflecting an increasing z-score).

Characterization of the sulco-gyral anatomy was assessed using a four-step procedure (Fig 1) with Morphologist 2012, a toolbox of BrainVisa software (http://brainvisa.info): (1) extraction of cortical folds; (2) automated recognition of standard sulci; (3) sulcus energy map generation, sulcus energy being a new criterion quantifying the degree of abnormality of cortical sulci [20]; and (4) computation of sulcus energy z-score maps, taking into account the variability of sulcus energy in controls (Fig 1B).

#### Cortical fold extraction

Cortical folds were automatically extracted from T1-weighted MRI including the following sub-steps: (i) segmentation of main brain tissues: cerebrospinal fluid, gray and white matter [25]; (ii) extraction of gray–white matter interface, and gray matter and cerebrospinal fluid interface and generation of associated surfaces; (iii) fold extraction from the skeleton of the gray matter/cerebrospinal fluid mask. The final extracted folds were converted to a graph-based representation of the cortex containing for each fold a list of morphological descriptors (area, depth, length, etc.) and spatial organization relative to neighbors (position, orientation) [26].

#### Automated sulci recognition

For each brain, sulci recognition was achieved through labeling of the folds with an anatomical nomenclature. It was performed using an automated algorithm, based on a congregation of artificial neural networks trained on a learning database [26,27]. The automated labeling followed an energy minimization performed using simulated annealing. In order to ensure the reliability of the labeling, ten automated recognitions were performed. The recognition with the lowest central sulcus/central region energy was chosen (for group/individual analysis respectively). This energy encodes a quantification of the similarities with the learning database. Hence, we made the hypothesis that the final minimal energy would be higher in FCD patients than in controls. In this study, we focused on this criterion without taking into account the accuracy of the recognition. For each subject, the final minimal energy reflects the best match with the learning database and provides a quantitative value of the matching quality, despite recognition errors in comparison with a manual recognition.

#### Sulcus energy map

For each subject, a sulcus energy map was generated from the above final minimal energy. It was split into a set of local energies for each sulcus depending on the sulcus itself and its relationship with its neighboring sulci. For the computation of this energy, several types of relations were taken into account: topological junction, neighbor geodesic to the brain hull, or split induced by a buried gyrus. A set of attributes was associated with each relation (eg. mean direction of the contact line between the two sulci, volume of the voxels involved in the relation, minimum distance between the two sulci, depth of the buried gyrus)[25,26,28]. Consequently, sulcus energy reflected not only the morphology of a given sulcus but also the pattern of the surrounding sulci. These local energies reflected the quality of the recognition: low local sulcus energy meant that the pattern of the sulcus and its neighbors matched the patterns of the learning database, whereas high local sulcus energy meant poor matching. Local sulcus energy therefore provides a new way to characterize abnormal sulco-gyral patterns.

#### Sulcus energy z-score map

In order to compare energy values between sulci, we normalized the sulcus energy map by taking into account the normal variability of each sulcus in controls [29,30]. Thus, for a given patient, we computed a z-score by dividing the difference between the sulcus energy of the patient and the mean sulcus energy of controls by the standard deviation of controls (Fig 1B). A z-score was computed sulcus by sulcus.

### Visual inspection

**Fig 2:**
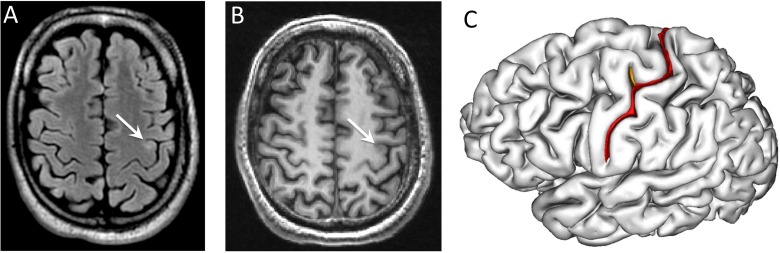
Visual identification of FCD sulci in a MR+ patient. The hyperintensities in FLAIR (A) and the slight cortical thickening visible in T1 MRI (B) allowed to locate the lesion in the depth of an ascending branch of the left central sulcus (white arrows), at the intersection of the main branch of the central sulcus. These two sulci are automatically labeled (C): « superior branch of the pre-central sulcus» (orange) and « central sulcus » (red) and considered as FCD sulci.

**Fig 3:**
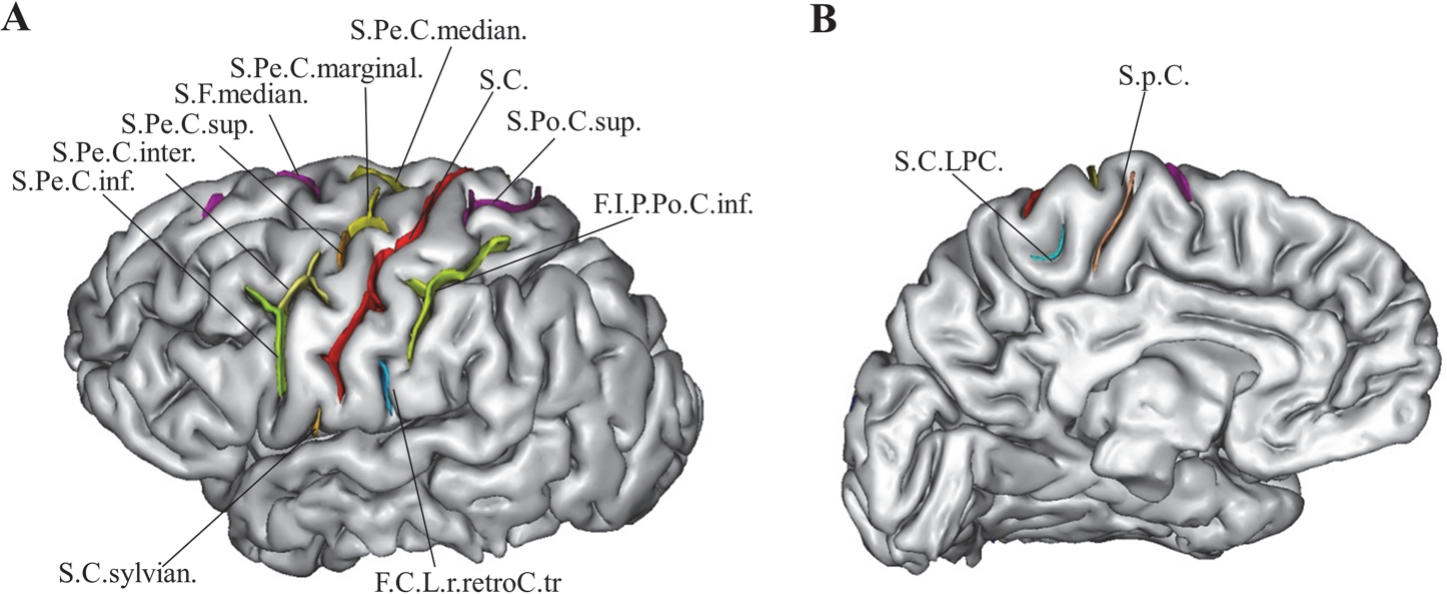
Sulci of the left central area, based on BrainVisa nomenclature (http://brainvisa.info), lateral (A) and medial (B) views: The central sulcus (S.C.) is surrounded by the superior postcentral sulcus (S.Po.C.sup), the retrocentral transverse ramus of the lateral fissure (F.C.L.r.retroC.tr), the inferior postcentral ramus of the intraparietal sulcus (F.I.P.Po.C.inf), the median frontal sulcus (S.F.median) and the median (S.Pe.C.median), marginal (S.Pe.C.marginal), superior (S.Pe.C.sup.), intermediate (S.Pe.C.inter.) and inferior (S.Pe.C.inf.) branches of the pre-central sulcus. The ramifications of the central sulcus were composed of the central sylvian sulcus (S.C.sylvian), the paracentral lobule (S.C.LPC.) and the paracentral sulcus (S.p.C). Right hemisphere not shown.

MRIs were reviewed retrospectively by an experienced neuroradiologist (C.M., 9 years’ experience in epilepsy MRI) blind to the sulco-gyral analysis but aware of all presurgical evaluation data, including PET. He was asked to look for four typical FCD MRI criteria [3]: (1) cortical thickening; (2) abnormal cortical signal intensity; (3) blurring of the gray–white matter junction; (4) abnormal signal intensity of subcortical and deep white matter. MR imaging was considered as positive (MR+) for the diagnosis of FCD when at least one of these criteria was present and as MR- otherwise. Each gray–white matter interface generated by BrainVisa was visually inspected and manually corrected if necessary. All labeled sulci were then reviewed by an expert (F.C. or C.M.), who identified the cortical sulci involved in the FCD (hereafter referred as the FCD sulci). “FCD sulci” included all automatically labeled sulci facing MR abnormalities in MR+ patients (Fig 2) or based on complete presurgical evaluation (including PET and stereo-EEG) and surgical data confirmed by histology in MR- patients. The nomenclature of the sulci of the central region provided by BrainVisa is defined in Fig 3.

### Statistical analyses

#### Group-wise analysis

Group-wise statistical analyses were performed with R software (http://www.r-project.org). The effect was considered as significant for p-values lower than 0.05.

#### Global FCD effect on central sulci

In order to assess a global effect of FCD on the sulcal morphology, we compared energies of the central sulci between patients and controls using multivariate analysis of variance (MANOVA) with repeated measures for both hemispheres, followed by post hoc comparisons. To correct for potential confounding effects of age [31], gender [32,33], and MR field on the sulco-gyral anatomy, these covariates were added in the linear models.

#### FCD effect on ipsilateral and contralateral central sulci

In order to investigate whether FCD had an effect on sulcal patterns ipsilateral or contralateral to the lesion, we performed additional analyses. We first compared energies of the central sulcus ipsilateral to the lesion between patients and controls (ANOVA with age, gender, MR field and hemisphere as cofactors). We then compared energies of the central sulcus contralateral to the lesion between patients and controls (ANOVA with the same cofactors as previously).

#### Individual analysis: Identification of FCD sulci in patients

In order to assess the relevance of this sulcus energy z-score map to localize the FCD, we computed the proportion of patients in whom an FCD sulcus corresponded to the maximum of z-score (the most abnormal sulcus relative to the variability observed in controls, Fig 1B). Given that the electroclinical characteristics of partial seizures originated from the central region typically point to one hemisphere, we restricted the analysis to the central region ipsilateral to the FCD. The significance of these results was tested using a permutation test (10 000 permutations) on the characteristics of sulci (FCD or not) and the p-value was set to 0.05 for statistical significance.

For comparison with a previous study [23], we also evaluated the ability of the sulcus energy z-score to identify FCD sulci by computing sulcus-wise sensitivity and specificity with various z-score thresholds. True positives and false negatives were defined as above-threshold and below-threshold FCD sulci, respectively; false positives and true negatives were defined respectively as above-threshold and below-threshold sulci outside the FCD sulci subset.

## Results

### Study groups—Visual MR analysis

FCD lesions were visually identified in 17 of the 29 (57%) patients and composed the MR+ subgroup. The segmentation was successful in all but three subjects, for whom a manual intervention excluded small parts of longitudinal venous sinus that were misclassified as gray matter. In the whole of the patient group, a total of 45 FCD sulci were identified, the lesion being in the vicinity of several neighboring sulci in some patients. The FCD lesions were preferentially located on either the central sulcus (eight patients) or the marginal precentral sulcus (eight patients). The sulci involved in the remaining cases were the intermediate (five patients), superior (four patients) and inferior (four patients) branches of the precentral sulcus, the central branch of the median frontal sulcus (four patients), the paracentral sulcus (two patients), the paracentral lobule sulcus (three patients) and the median branch of the precentral sulcus (four patients), the central sylvian sulcus (two patients) and the inferior postcentral ramus of the intraparietal sulcus (one patient). A comprehensive list of FCD sulci for each patient can be found in Table 2. The demographics and the FCD type of the MR+ and MR- subgroups are detailed in Table 3. These characteristics were similar between subgroups except for age (p = 0.02), related to a bias of recruitment for pediatric MR- cases.

**Table 2:** List of FCD sulci for each patient.

	MR group	lesion side	number of FCD sulci	list of FCD sulci
1	MR-	left	1	S.C.LPC.
2	MR-	left	1	S.C.
3	MR-	left	1	S.F.median.
4	MR-	left	1	S.C.
5	MR-	right	1	S.Pe.C.marginal.
6	MR-	right	2	S.C., S.Pe.C.inter.
7	MR-	right	2	S.Pe.C.inf., S.Pe.C.inter.
8	MR-	right	1	F.I.P.Po.C.inf.
9	MR-	right	2	S.Pe.C.marginal.,t S.Pe.C.median.,
10	MR-	right	1	S.Pe.C.median.,
11	MR-	right	3	S.C., S.Pe.C.sup., S.Pe.C.inter.
12	MR+	left	1	S.Pe.C.inter.,
13	MR+	left	2	S.Pe.C.median., S.Pe.C.marginal.
14	MR+	left	2	S.Pe.C.marginal., S.F.median.
15	MR+	left	3	S.p.C., S.C.LPC., S.F.median.
16	MR+	left	1	S.Pe.C.sup.
17	MR+	left	1	S.Pe.C.marginal.
18	MR+	left	3	S.Pe.C.inf., S.Pe.C.inter., S.C.sylvian.
19	MR+	left	2	S.p.C., S.C.LPC.
20	MR+	left	3	S.Pe.C.sup., S.C., S.Pe.C.marginal.
21	MR+	right	3	S.Pe.C.marginal., S.Pe.C.sup., S.Pe.C.median.
22	MR+	right	1	S.Pe.C.marginal.
23	MR+	right	1	S.F.median.
24	MR+	right	2	S.Pe.C.inf., S.C.sylvian.
25	MR+	right	1	S.Pe.C.inf.
26	MR+	right	1	S.C.
27	MR+	right	1	S.C.
28	MR+	right	1	S.C.

**Table 3:** Demographic data of MR+ and MR- patient subgroups.

	MR+	MR-
Number	17	12
Males (%)	11 (65)	4 (33)
Age: median (IQR)	24 (19–37)	14 (11–18)
Histologically proven FCD	13	12
FCD type	13 IIB	9 IIB, 3 IIA
Side of the FCD (%)	8 Right (47)	7 Right (58)

IQR: interquartile range.

### Group-wise statistical analysis

#### Global FCD effect on central sulci

Patients had significantly higher central sulcus energy than controls (p = 0.006), whereas the main effect of sex (p = 0.06), age (p = 0.12) and MR field (p = 0.07) was not significant. Similarly, when considered separately, MR+ (p = 0.033) and MR- patients (p = 0.034) had higher central sulcus energy than controls.

#### FCD effect on ipsilateral and contralateral central sulci

Patients had higher central sulcus energy ipsilateral to the FCD compared to the central sulcus in controls (p = 0.032), whereas the main effect of sex (p = 0.531), age (p = 0.683), MR field (p = 0.161) and lesion side (p = 0.195) was not significant. The same result was found when only MR+ patients were compared with controls (p = 0.038) but not when MR- patients were compared with controls (p = 0.186). The central sulcus energy contralateral to the lesion did not differ between patients and controls (p = 0.192).

### Individual analysis

**Fig 4:**
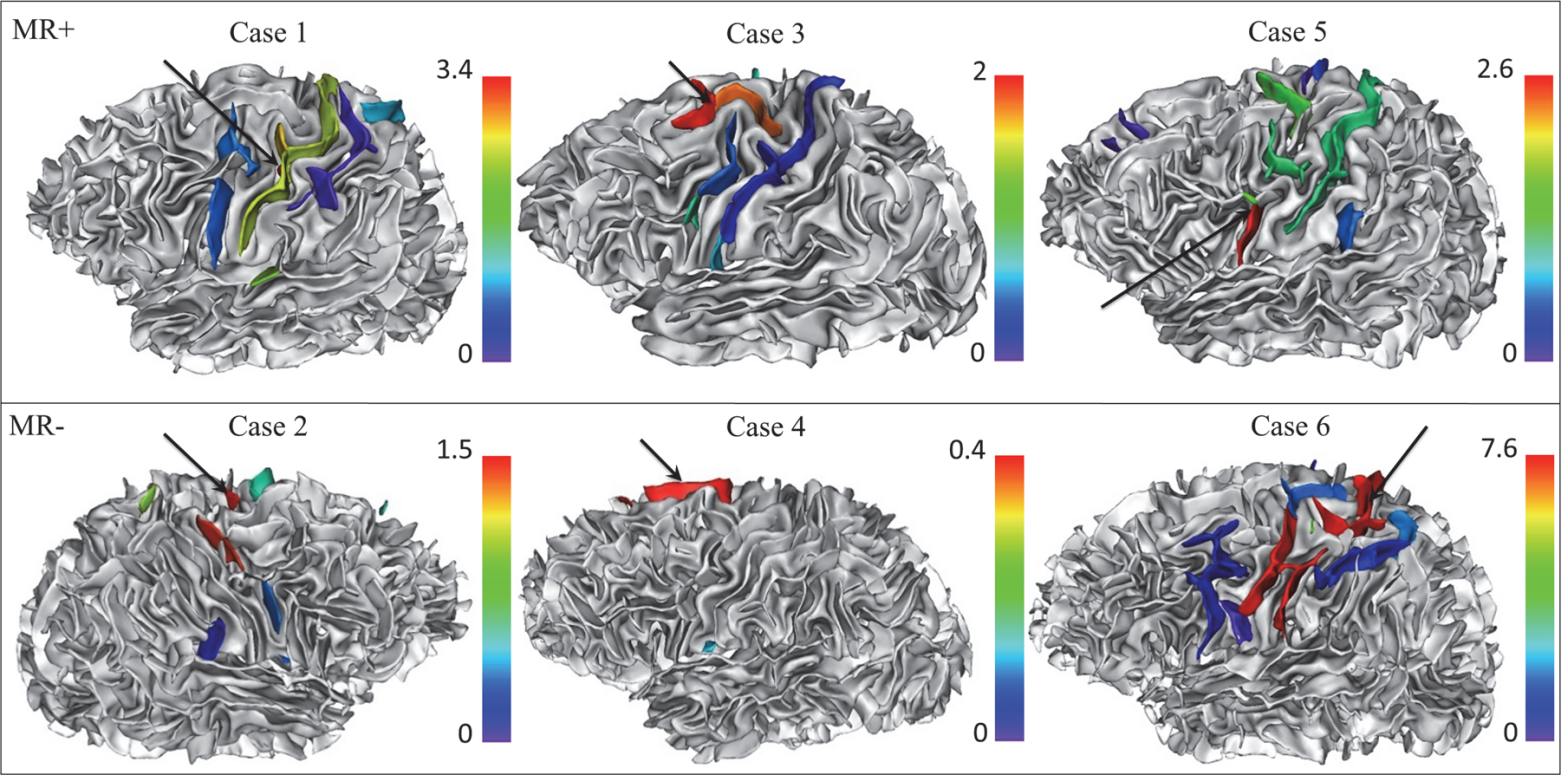
Illustrative examples of patients with maximum z-scores pointing to an FCD sulcus. Z-score maps superimposed on gray–white matter interface surface models. For each patient, a color palette from blue to red was adjusted to the maximum z-score. Black arrow indicated the lesion. In Case 1, the maximum z-score (z = 3.4) pointed exactly to the FCD (red blob, arrow), in the depth of an ascending branch of the left central sulcus. In Case 2, the lesion was located on the right marginal precentral sulcus, which had a maximum z-score of 1.52. Of note, the right superior precentral sulcus (in red) also had a high, albeit not maximum, z-score (1.49). In Cases 3 and 4, the maximum z-scores (z = 2 and 0.4 respectively) pointed to the FCD sulcus, which stood-out from the neighboring sulci with much lower z-scores. In Case 5, the maximum z-score (z = 2.6) pointed to an FCD sulcus but there were other sulci with high local z-score beyond the FCD. In Case 6, even if the FCD was associated with a maximum z-score (z = 7.6), this high energy sulcus had a wide spatial extent, beyond the site of FCD.

**Fig 5:**
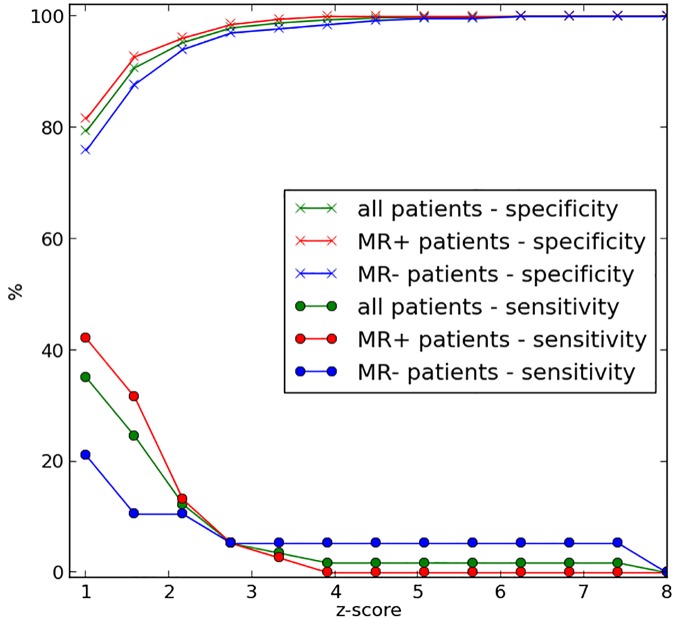
Sensitivity and specificity for quantitatively derived abnormal sulci compared with FCD sulci.

In controls, the mean sulcus energy differed across the 13 sulci of the central region (Fig 1) with expected sulcus energies reflecting the degree of anatomical variability: for instance, the central sulcus, known to be the most stable sulcus across individuals, had the lowest mean energy whereas the central sylvian sulcus, a small branch of the central sulcus, had the highest mean energy. Fig 4 shows examples of sulcus energy z-score maps for six patients taking into account the above-mentioned normal inter-sulcus variability in controls. For patients, the analysis relied on 28 patients as in one MR- case, the FCD sulcus was mislabeled as outside the central region. In nine (four MR- and five MR+) of 28 patients (32%), the maximum z-score pointed to an FCD sulcus (p = 0.0046). When MR+ and MR- patients were analyzed separately, these results remained significant for MR- patients (p = 0.024) but not for MR+ patients (p = 0.058). Fig 5 presents sensitivity and specificity curves related to the localization of FCD sulci in patients for different energy z-score thresholds. The area under the receiver operating characteristic curve was similar for MR+ and MR- patients (0.611 and 0.606, respectively).

## Discussion

We found abnormal sulcal patterns in patients with FCD located in the central region after correction for potential bias factors such as gender, age and MR field strength. This group finding provides proof of concept for studying sulcal abnormalities in FCD patients, even if these are not yet considered as a cardinal sign of FCD. At the individual level, the automated sulcal energy analysis we used takes into account normal interindividual sulcal variability through the use of a group of healthy controls. Our findings strengthen the previously reported association between subclinical abnormal sulcal patterns and the epileptogenic zone in MR- frontal FCD patients based on visual inspection of sulcus energy maps [20].

In the group-wise comparison, patients with FCD had abnormal sulcal patterns of the central regions. This was observed for the whole FCD population and when MR+ and MR- patients were considered separately. Our results are consistent with the greater depth of FCD sulci compared with the corresponding sulci in controls reported in a previous study [22]. Our results also match the visually detectable sulcal abnormalities in FCD reported in several previous studies [3,6,7,34]. One study noted that, even if present in MR- patients, visually detectable unusual sulcal patterns were more prominent in MR+ than in MR- patients [3]. This may explain why sulcal patterns of the central sulcus ipsilateral to the FCD differed from controls for MR+ but not for MR- patients in our study.

Other results of the individual analysis deserve attention. Although sulcus energy variability was present in controls, we found a link between the location of the FCD and the sulcus energy z-scores. Indeed, the proportion of patients for whom the maximum energy z-scores identified an FCD sulcus was above the chance level. In line with this, the fact that the maximum sulcus energy z-scores pointed to the lesion in 4 of the 11 MR- cases is promising and clinically relevant as it could increase the number of candidates for surgery. These results encourage future studies combining the sulcus energy z-score map with other presurgical data, as previously done with voxel-based morphometry [14]. However, the sulcus energy z-score map alone was not sufficient to identify the FCD sulci. Indeed, the maximum z-score pointed to a sulcus distant from FCD in 67% of patients and we did not find a z-score threshold allowing a good trade-off between sensitivity/specificity in order to achieve a satisfactory level of FCD detection. This ties in with a previous study [23], which reported that surface-based morphometry analysis failed to adequately describe lesion extent in patients with epileptogenic malformations. The presence of these false positives distant from the FCD is consistent with other imaging studies reporting extra-lesional abnormalities such as an abnormal sulcal pattern [20] or gray matter increase [13]. It is also consistent with the developmental origin of FCD. Indeed, FCD is a disorder occurring very early in brain morphogenesis [35], especially during the development of cortical sulci. Although the mechanisms of this sulcogenesis remain poorly understood, different models have been proposed [36–38]. Among them, the theory of tension-based morphogenesis [36] postulated that sulcogenesis relies on the global minimization over the brain of the tensions from white matter fibers connecting cortical areas. This link between connectivity and sulcal patterns is supported by experimental data indicating that alterations of thalamo-cortical or cortico-cortical connections affected the gyral patterns [39]. In this context, the extra-lesional high sulcus energy z-scores we observed are not surprising considering that recent results using diffusion MRI have also shown bilateral and widespread patterns of white matter microstructural abnormalities extending beyond the FCD lesion [40]. Multimodal studies combining conventional or advanced diffusion techniques [41] and morphological MR imaging data may bring further insight into the link between sulcal and anatomical connectivity impairments related to FCD.

Our study suffers from several limitations. First, our findings cannot currently be applied to left-handed patients, whereas a substantial proportion of patients with FCD are left-handed. In our center, although 24% of patients with FCD of the central region were left-handed, we chose to focus our study on right-handed subjects to avoid the confounding effect of handedness. Indeed, a recent study demonstrated that sinistrals differ from dextrals in the shape of certain cortical folds, especially the central sulcus [42]. In addition, in the Morphologist toolbox of BrainVisa, the learning database of healthy subjects was composed solely of right-handed subjects. Second, in the current study, 76% of patients were imaged on a 1.5T MR, which might have been less sensitive than 3T for the detection of FCD. However, we have previously shown that 3T MRI only marginally increases the number of FCD detected as compared with 1.5T when using a similar acquisition time [43]. To minimize the unlikely influence of the magnetic field on our results, we also considered this MR field strength as a cofactor in the group-wise statistical analysis. Third, the brain segmentation could potentially be perturbed by signal intensity changes associated with the FCD, which could impact the reconstruction of the underlying sulci. However, we systematically controlled the segmentation results and did not identify any such disturbances. As opposed to other methods, such as voxel-based or surface-based morphometry, our methodology does not require registration between the individual subjects and is therefore free from the potential effects of misregistration due to the lesion. Fourth, the sulcus-based method we used was not designed to detect epileptogenic lesions, but rather to automatically recognize cortical folds, and especially the sulcus energy. New tools have been proposed to characterize and quantify the shape variability of a single sulcus, based on shape similarity measurements and on manifold learning [42]. Combining such new techniques and clinical a priori knowledge could be of great help in improving the methodology.

## Conclusion

In this study, we demonstrated the ability of an automated sulcus energy-based analysis to identify abnormal sulcal patterns in patients with FCD of the central region compared with healthy controls. The abnormal patterns of the central sulcus ipsilateral to the FCD and the link between maximum sulcus energy z-scores and the site of FCD strengthen the importance of studying sulcal patterns in FCD patients. The individual sulcus-based analysis pointed to the FCD site in almost half of patients with normal MRI, a finding that has clinical relevance: combining the sulcus energy z-score map with other presurgical data may in future help to localize FCD lesions in patients with epilepsy of the central region.
